# Supplementary material for: Modeling glioblastoma heterogeneity as a dynamic network of cell states
Source: Mol Syst Biol. 2021 Sep 16;17(9):e10105. doi: 10.15252/msb.202010105 (PMC8444284; doi:10.15252/msb.202010105)
Supplement: Supplementary file 5 — Source Data for Figure 3 [file MSB-17-e10105-s001.zip › Figure3A_sourcedata/GSEA_3065/nosigdb_state2.GseaPreranked.1622718419215/VANGRONINGEN_MES.html]

Details for gene set VANGRONINGEN\_MES[GSEA]

|  || Dataset | state2 |
| Phenotype | NoPhenotypeAvailable |
| Upregulated in class | na\_neg |
| GeneSet | VANGRONINGEN\_MES |
| Enrichment Score (ES) | -0.6593999 |
| Normalized Enrichment Score (NES) | -2.9422407 |
| Nominal p-value | 0.0 |
| FDR q-value | 0.0 |
| FWER p-Value | 0.0 |
Table: GSEA Results Summary

  

Fig 1: Enrichment plot: VANGRONINGEN\_MES      
 Profile of the Running ES Score & Positions of GeneSet Members on the Rank Ordered List

  

| PROBE | GENE SYMBOL | GENE\_TITLE | RANK IN GENE LIST | RANK METRIC SCORE | RUNNING ES | CORE ENRICHMENT || 1 | MYL12B |  |  | 383 | 0.208 | -0.0371 | No |
| 2 | PDLIM1 |  |  | 452 | 0.190 | -0.0398 | No |
| 3 | NFIC |  |  | 543 | 0.174 | -0.0454 | No |
| 4 | TUBB6 |  |  | 622 | 0.160 | -0.0500 | No |
| 5 | RAP1B |  |  | 806 | 0.137 | -0.0668 | No |
| 6 | SEPT10 |  |  | 869 | 0.131 | -0.0704 | No |
| 7 | PTBP1 |  |  | 943 | 0.124 | -0.0753 | No |
| 8 | DESI2 |  |  | 1055 | 0.115 | -0.0847 | No |
| 9 | ELAVL1 |  |  | 1122 | 0.110 | -0.0893 | No |
| 10 | MBD2 |  |  | 1136 | 0.109 | -0.0880 | No |
| 11 | GNG12 |  |  | 1154 | 0.108 | -0.0872 | No |
| 12 | NANS |  |  | 1289 | 0.099 | -0.0995 | No |
| 13 | DUSP6 |  |  | 1345 | 0.095 | -0.1032 | No |
| 14 | MOB1A |  |  | 1400 | 0.092 | -0.1068 | No |
| 15 | UAP1 |  |  | 1401 | 0.092 | -0.1045 | No |
| 16 | SOX9 |  |  | 1466 | 0.088 | -0.1094 | No |
| 17 | CMTM3 |  |  | 1550 | 0.084 | -0.1165 | No |
| 18 | SPRY1 |  |  | 1623 | 0.081 | -0.1224 | No |
| 19 | PXDC1 |  |  | 1634 | 0.080 | -0.1215 | No |
| 20 | SSBP4 |  |  | 1662 | 0.079 | -0.1225 | No |
| 21 | LITAF |  |  | 1685 | 0.078 | -0.1230 | No |
| 22 | EGR1 |  |  | 1691 | 0.077 | -0.1216 | No |
| 23 | TJP1 |  |  | 1811 | 0.072 | -0.1329 | No |
| 24 | PLSCR1 |  |  | 1853 | 0.070 | -0.1357 | No |
| 25 | SYDE1 |  |  | 1906 | 0.068 | -0.1398 | No |
| 26 | HOMER1 |  |  | 1912 | 0.068 | -0.1386 | No |
| 27 | FAM129A |  |  | 1946 | 0.067 | -0.1406 | No |
| 28 | RAB31 |  |  | 1977 | 0.065 | -0.1423 | No |
| 29 | FAM120A |  |  | 2030 | 0.063 | -0.1464 | No |
| 30 | MEOX2 |  |  | 2062 | 0.062 | -0.1483 | No |
| 31 | CBFB |  |  | 2143 | 0.060 | -0.1556 | No |
| 32 | PEA15 |  |  | 2161 | 0.059 | -0.1560 | No |
| 33 | RHOC |  |  | 2261 | 0.055 | -0.1656 | No |
| 34 | ZFP36L1 |  |  | 2320 | 0.053 | -0.1707 | No |
| 35 | STAT1 |  |  | 2336 | 0.053 | -0.1710 | No |
| 36 | CNN3 |  |  | 2349 | 0.052 | -0.1710 | No |
| 37 | KANK2 |  |  | 2413 | 0.050 | -0.1767 | No |
| 38 | HIBADH |  |  | 2432 | 0.050 | -0.1775 | No |
| 39 | POLR2L |  |  | 2435 | 0.050 | -0.1765 | No |
| 40 | CETN2 |  |  | 2466 | 0.049 | -0.1786 | No |
| 41 | ARL4A |  |  | 2510 | 0.047 | -0.1821 | No |
| 42 | PHTF2 |  |  | 2622 | 0.044 | -0.1933 | No |
| 43 | KDELC2 |  |  | 2632 | 0.043 | -0.1932 | No |
| 44 | SPRY4 |  |  | 2656 | 0.043 | -0.1947 | No |
| 45 | ZCCHC24 |  |  | 2705 | 0.041 | -0.1989 | No |
| 46 | NFIA |  |  | 2796 | 0.039 | -0.2079 | No |
| 47 | ANXA5 |  |  | 2812 | 0.039 | -0.2086 | No |
| 48 | RAP1A |  |  | 2848 | 0.038 | -0.2115 | No |
| 49 | SEC14L1 |  |  | 2876 | 0.037 | -0.2135 | No |
| 50 | UGDH |  |  | 2897 | 0.036 | -0.2148 | No |
| 51 | RNH1 |  |  | 2962 | 0.035 | -0.2210 | No |
| 52 | ETS1 |  |  | 2998 | 0.034 | -0.2241 | No |
| 53 | SUCLG2 |  |  | 3045 | 0.033 | -0.2283 | No |
| 54 | MYL12A |  |  | 3048 | 0.033 | -0.2277 | No |
| 55 | IFI16 |  |  | 3054 | 0.033 | -0.2275 | No |
| 56 | SDCBP |  |  | 3058 | 0.033 | -0.2270 | No |
| 57 | GAS1 |  |  | 3144 | 0.031 | -0.2356 | No |
| 58 | ARL1 |  |  | 3309 | 0.027 | -0.2530 | No |
| 59 | OGFRL1 |  |  | 3489 | 0.024 | -0.2722 | No |
| 60 | HSPB1 |  |  | 3629 | 0.022 | -0.2870 | No |
| 61 | PRDX6 |  |  | 3639 | 0.022 | -0.2874 | No |
| 62 | SIX1 |  |  | 3649 | 0.021 | -0.2879 | No |
| 63 | ENAH |  |  | 3666 | 0.021 | -0.2891 | No |
| 64 | PLEKHA2 |  |  | 3822 | 0.018 | -0.3058 | No |
| 65 | RAB13 |  |  | 3928 | 0.017 | -0.3169 | No |
| 66 | TSPAN4 |  |  | 3929 | 0.017 | -0.3165 | No |
| 67 | ABRACL |  |  | 4045 | 0.015 | -0.3288 | No |
| 68 | EXT1 |  |  | 4083 | 0.014 | -0.3326 | No |
| 69 | ARHGEF40 |  |  | 4089 | 0.014 | -0.3328 | No |
| 70 | SPRED1 |  |  | 4165 | 0.013 | -0.3407 | No |
| 71 | ACAP2 |  |  | 4173 | 0.013 | -0.3412 | No |
| 72 | YAP1 |  |  | 4184 | 0.012 | -0.3420 | No |
| 73 | DUSP14 |  |  | 4320 | 0.010 | -0.3566 | No |
| 74 | TOR1AIP1 |  |  | 4475 | 0.008 | -0.3735 | No |
| 75 | PIAS3 |  |  | 4491 | 0.007 | -0.3749 | No |
| 76 | TGFB1I1 |  |  | 4531 | 0.007 | -0.3791 | No |
| 77 | IQGAP2 |  |  | 4559 | 0.006 | -0.3819 | No |
| 78 | SMAD3 |  |  | 4579 | 0.006 | -0.3838 | No |
| 79 | ANXA6 |  |  | 4580 | 0.006 | -0.3837 | No |
| 80 | RGS10 |  |  | 4655 | 0.005 | -0.3917 | No |
| 81 | RAB29 |  |  | 4671 | 0.005 | -0.3933 | No |
| 82 | ELK3 |  |  | 4675 | 0.004 | -0.3935 | No |
| 83 | CLIC4 |  |  | 4778 | 0.003 | -0.4047 | No |
| 84 | TFE3 |  |  | 4824 | 0.002 | -0.4096 | No |
| 85 | PYGL |  |  | 4875 | 0.001 | -0.4151 | No |
| 86 | ERLIN1 |  |  | 4947 | 0.001 | -0.4229 | No |
| 87 | AMMECR1 |  |  | 4983 | 0.000 | -0.4268 | No |
| 88 | SASH1 |  |  | 4986 | 0.000 | -0.4270 | No |
| 89 | PHLDB2 |  |  | 5023 | -0.000 | -0.4309 | No |
| 90 | JAK1 |  |  | 5116 | -0.002 | -0.4410 | No |
| 91 | EPS8 |  |  | 5190 | -0.003 | -0.4490 | No |
| 92 | TCF7L2 |  |  | 5199 | -0.003 | -0.4498 | No |
| 93 | S1PR3 |  |  | 5237 | -0.004 | -0.4538 | No |
| 94 | ELF1 |  |  | 5246 | -0.004 | -0.4546 | No |
| 95 | KLF10 |  |  | 5308 | -0.005 | -0.4612 | No |
| 96 | LIX1L |  |  | 5440 | -0.007 | -0.4755 | No |
| 97 | SKIL |  |  | 5485 | -0.008 | -0.4801 | No |
| 98 | FAM114A1 |  |  | 5495 | -0.008 | -0.4809 | No |
| 99 | GNAI1 |  |  | 5503 | -0.009 | -0.4815 | No |
| 100 | NID2 |  |  | 5511 | -0.009 | -0.4820 | No |
| 101 | NID1 |  |  | 5537 | -0.009 | -0.4846 | No |
| 102 | ELK4 |  |  | 5564 | -0.009 | -0.4872 | No |
| 103 | RHOJ |  |  | 5586 | -0.010 | -0.4893 | No |
| 104 | L3HYPDH |  |  | 5589 | -0.010 | -0.4893 | No |
| 105 | PALLD |  |  | 5615 | -0.010 | -0.4918 | No |
| 106 | TXNDC12 |  |  | 5696 | -0.011 | -0.5003 | No |
| 107 | SFT2D2 |  |  | 5756 | -0.012 | -0.5065 | No |
| 108 | INSIG1 |  |  | 5815 | -0.013 | -0.5126 | No |
| 109 | GORAB |  |  | 5816 | -0.013 | -0.5122 | No |
| 110 | FZD1 |  |  | 5844 | -0.014 | -0.5149 | No |
| 111 | STK38L |  |  | 5935 | -0.015 | -0.5244 | No |
| 112 | REST |  |  | 5962 | -0.016 | -0.5269 | No |
| 113 | CRTAP |  |  | 5980 | -0.016 | -0.5284 | No |
| 114 | CSRP1 |  |  | 6030 | -0.017 | -0.5333 | No |
| 115 | CCDC80 |  |  | 6077 | -0.018 | -0.5380 | No |
| 116 | MBNL1 |  |  | 6105 | -0.018 | -0.5405 | No |
| 117 | GPX8 |  |  | 6123 | -0.019 | -0.5419 | No |
| 118 | IFITM2 |  |  | 6128 | -0.019 | -0.5419 | No |
| 119 | HES1 |  |  | 6191 | -0.020 | -0.5482 | No |
| 120 | C6orf120 |  |  | 6260 | -0.021 | -0.5552 | No |
| 121 | LASP1 |  |  | 6308 | -0.022 | -0.5599 | No |
| 122 | SNAP23 |  |  | 6326 | -0.022 | -0.5612 | No |
| 123 | CRISPLD1 |  |  | 6341 | -0.022 | -0.5622 | No |
| 124 | ARHGAP1 |  |  | 6345 | -0.022 | -0.5619 | No |
| 125 | CYFIP1 |  |  | 6368 | -0.023 | -0.5638 | No |
| 126 | SFT2D1 |  |  | 6393 | -0.023 | -0.5659 | No |
| 127 | ERRFI1 |  |  | 6445 | -0.024 | -0.5709 | No |
| 128 | NES |  |  | 6455 | -0.025 | -0.5713 | No |
| 129 | HS3ST3A1 |  |  | 6499 | -0.025 | -0.5754 | No |
| 130 | SH3BGRL |  |  | 6546 | -0.026 | -0.5798 | No |
| 131 | ADGRG6 |  |  | 6559 | -0.026 | -0.5805 | No |
| 132 | DCAF6 |  |  | 6586 | -0.027 | -0.5827 | No |
| 133 | BTN3A2 |  |  | 6686 | -0.029 | -0.5929 | No |
| 134 | TRIM5 |  |  | 6780 | -0.031 | -0.6024 | No |
| 135 | NR3C1 |  |  | 6802 | -0.032 | -0.6039 | No |
| 136 | CTDSP2 |  |  | 6841 | -0.032 | -0.6073 | No |
| 137 | SLC30A7 |  |  | 6878 | -0.033 | -0.6104 | No |
| 138 | GALNT10 |  |  | 6881 | -0.034 | -0.6098 | No |
| 139 | EFEMP2 |  |  | 6895 | -0.034 | -0.6104 | No |
| 140 | RECK |  |  | 6921 | -0.035 | -0.6123 | No |
| 141 | SLC38A6 |  |  | 6935 | -0.035 | -0.6128 | No |
| 142 | SLC30A1 |  |  | 6951 | -0.035 | -0.6136 | No |
| 143 | MGAT2 |  |  | 6959 | -0.035 | -0.6135 | No |
| 144 | KLF6 |  |  | 7010 | -0.037 | -0.6181 | No |
| 145 | MANF |  |  | 7013 | -0.037 | -0.6174 | No |
| 146 | PEAK1 |  |  | 7039 | -0.038 | -0.6192 | No |
| 147 | LUZP1 |  |  | 7053 | -0.038 | -0.6197 | No |
| 148 | C1orf54 |  |  | 7100 | -0.039 | -0.6238 | No |
| 149 | CTSO |  |  | 7105 | -0.039 | -0.6233 | No |
| 150 | AEBP1 |  |  | 7162 | -0.041 | -0.6284 | No |
| 151 | DSE |  |  | 7207 | -0.042 | -0.6322 | No |
| 152 | EHD2 |  |  | 7220 | -0.043 | -0.6325 | No |
| 153 | TRAM2 |  |  | 7251 | -0.043 | -0.6347 | No |
| 154 | TMEM263 |  |  | 7263 | -0.044 | -0.6348 | No |
| 155 | ANXA1 |  |  | 7265 | -0.044 | -0.6339 | No |
| 156 | ITPRIPL2 |  |  | 7268 | -0.044 | -0.6330 | No |
| 157 | CTNNA1 |  |  | 7279 | -0.044 | -0.6330 | No |
| 158 | NBR1 |  |  | 7303 | -0.045 | -0.6344 | No |
| 159 | CMTM6 |  |  | 7313 | -0.045 | -0.6343 | No |
| 160 | HIPK3 |  |  | 7332 | -0.045 | -0.6351 | No |
| 161 | ZNF217 |  |  | 7333 | -0.045 | -0.6340 | No |
| 162 | TWSG1 |  |  | 7335 | -0.045 | -0.6330 | No |
| 163 | KDELR3 |  |  | 7339 | -0.046 | -0.6322 | No |
| 164 | ACTN1 |  |  | 7366 | -0.046 | -0.6339 | No |
| 165 | RGL1 |  |  | 7385 | -0.047 | -0.6347 | No |
| 166 | TPM1 |  |  | 7414 | -0.048 | -0.6366 | No |
| 167 | SHROOM3 |  |  | 7472 | -0.050 | -0.6416 | No |
| 168 | QKI |  |  | 7478 | -0.050 | -0.6409 | No |
| 169 | KIF13A |  |  | 7500 | -0.050 | -0.6420 | No |
| 170 | ID1 |  |  | 7519 | -0.051 | -0.6427 | No |
| 171 | EXTL2 |  |  | 7527 | -0.051 | -0.6422 | No |
| 172 | EDEM1 |  |  | 7566 | -0.052 | -0.6451 | No |
| 173 | GPR137B |  |  | 7585 | -0.053 | -0.6458 | No |
| 174 | PHLDA3 |  |  | 7590 | -0.053 | -0.6449 | No |
| 175 | HEXB |  |  | 7621 | -0.054 | -0.6468 | No |
| 176 | ACADVL |  |  | 7628 | -0.054 | -0.6462 | No |
| 177 | RBMS1 |  |  | 7667 | -0.055 | -0.6490 | No |
| 178 | TSC22D2 |  |  | 7695 | -0.056 | -0.6506 | No |
| 179 | ARPC1B |  |  | 7700 | -0.056 | -0.6496 | No |
| 180 | ADGRE5 |  |  | 7701 | -0.056 | -0.6482 | No |
| 181 | MAGT1 |  |  | 7724 | -0.057 | -0.6492 | No |
| 182 | CYBRD1 |  |  | 7725 | -0.057 | -0.6478 | No |
| 183 | FZD2 |  |  | 7754 | -0.058 | -0.6494 | No |
| 184 | IL13RA1 |  |  | 7762 | -0.058 | -0.6487 | No |
| 185 | FKBP14 |  |  | 7766 | -0.059 | -0.6476 | No |
| 186 | ARMCX2 |  |  | 7832 | -0.061 | -0.6532 | No |
| 187 | SPATA20 |  |  | 7859 | -0.062 | -0.6546 | No |
| 188 | LOXL2 |  |  | 7871 | -0.062 | -0.6542 | No |
| 189 | RIT1 |  |  | 7880 | -0.062 | -0.6536 | No |
| 190 | A2M |  |  | 7912 | -0.063 | -0.6554 | No |
| 191 | ATP10D |  |  | 7923 | -0.064 | -0.6549 | No |
| 192 | TNFRSF12A |  |  | 7931 | -0.064 | -0.6541 | No |
| 193 | SLC16A4 |  |  | 7964 | -0.066 | -0.6560 | No |
| 194 | LPP |  |  | 7981 | -0.066 | -0.6561 | No |
| 195 | DMD |  |  | 7987 | -0.067 | -0.6550 | No |
| 196 | DNAJC1 |  |  | 8012 | -0.068 | -0.6559 | No |
| 197 | TPM2 |  |  | 8019 | -0.068 | -0.6549 | No |
| 198 | DPY19L1 |  |  | 8021 | -0.068 | -0.6533 | No |
| 199 | FUCA2 |  |  | 8068 | -0.070 | -0.6566 | No |
| 200 | EGFR |  |  | 8094 | -0.071 | -0.6576 | Yes |
| 201 | BAG3 |  |  | 8104 | -0.071 | -0.6568 | Yes |
| 202 | FBN1 |  |  | 8107 | -0.072 | -0.6553 | Yes |
| 203 | SLC39A14 |  |  | 8119 | -0.072 | -0.6547 | Yes |
| 204 | LIFR |  |  | 8134 | -0.073 | -0.6544 | Yes |
| 205 | TM4SF1 |  |  | 8150 | -0.074 | -0.6542 | Yes |
| 206 | CAPN2 |  |  | 8165 | -0.075 | -0.6538 | Yes |
| 207 | SDC4 |  |  | 8172 | -0.076 | -0.6526 | Yes |
| 208 | LIPA |  |  | 8178 | -0.076 | -0.6513 | Yes |
| 209 | PLOD3 |  |  | 8187 | -0.077 | -0.6502 | Yes |
| 210 | SSR3 |  |  | 8191 | -0.077 | -0.6486 | Yes |
| 211 | ATP1B1 |  |  | 8199 | -0.077 | -0.6475 | Yes |
| 212 | SDF4 |  |  | 8241 | -0.079 | -0.6500 | Yes |
| 213 | CXCL12 |  |  | 8251 | -0.080 | -0.6490 | Yes |
| 214 | RNFT1 |  |  | 8284 | -0.082 | -0.6505 | Yes |
| 215 | PDGFC |  |  | 8287 | -0.082 | -0.6487 | Yes |
| 216 | NPC2 |  |  | 8297 | -0.083 | -0.6476 | Yes |
| 217 | SLC35F5 |  |  | 8314 | -0.084 | -0.6473 | Yes |
| 218 | FIBIN |  |  | 8317 | -0.084 | -0.6455 | Yes |
| 219 | KDELR2 |  |  | 8324 | -0.084 | -0.6440 | Yes |
| 220 | TRAM1 |  |  | 8333 | -0.085 | -0.6428 | Yes |
| 221 | CREB3L2 |  |  | 8341 | -0.085 | -0.6414 | Yes |
| 222 | NOTCH2 |  |  | 8358 | -0.086 | -0.6411 | Yes |
| 223 | OSTC |  |  | 8367 | -0.087 | -0.6398 | Yes |
| 224 | PPT1 |  |  | 8369 | -0.087 | -0.6377 | Yes |
| 225 | SHC1 |  |  | 8385 | -0.088 | -0.6372 | Yes |
| 226 | SPCS3 |  |  | 8396 | -0.089 | -0.6361 | Yes |
| 227 | P4HA1 |  |  | 8398 | -0.089 | -0.6340 | Yes |
| 228 | PLOD2 |  |  | 8403 | -0.089 | -0.6322 | Yes |
| 229 | PTPRK |  |  | 8411 | -0.090 | -0.6307 | Yes |
| 230 | CKAP4 |  |  | 8425 | -0.091 | -0.6299 | Yes |
| 231 | PRRX1 |  |  | 8427 | -0.091 | -0.6277 | Yes |
| 232 | SEL1L3 |  |  | 8436 | -0.091 | -0.6263 | Yes |
| 233 | MYADM |  |  | 8479 | -0.094 | -0.6286 | Yes |
| 234 | MAN2A1 |  |  | 8487 | -0.095 | -0.6270 | Yes |
| 235 | WIPI1 |  |  | 8505 | -0.096 | -0.6265 | Yes |
| 236 | MEST |  |  | 8534 | -0.099 | -0.6271 | Yes |
| 237 | GPC6 |  |  | 8558 | -0.100 | -0.6272 | Yes |
| 238 | OLFML2A |  |  | 8569 | -0.101 | -0.6257 | Yes |
| 239 | MBTPS1 |  |  | 8582 | -0.102 | -0.6245 | Yes |
| 240 | ATP2B1 |  |  | 8584 | -0.102 | -0.6221 | Yes |
| 241 | FILIP1L |  |  | 8586 | -0.102 | -0.6196 | Yes |
| 242 | COL11A1 |  |  | 8598 | -0.103 | -0.6183 | Yes |
| 243 | COPA |  |  | 8599 | -0.103 | -0.6157 | Yes |
| 244 | ITM2C |  |  | 8600 | -0.103 | -0.6131 | Yes |
| 245 | HYOU1 |  |  | 8614 | -0.104 | -0.6120 | Yes |
| 246 | CRELD2 |  |  | 8630 | -0.105 | -0.6110 | Yes |
| 247 | LMNA |  |  | 8636 | -0.106 | -0.6089 | Yes |
| 248 | SCPEP1 |  |  | 8654 | -0.107 | -0.6081 | Yes |
| 249 | GNS |  |  | 8677 | -0.109 | -0.6078 | Yes |
| 250 | LGALS1 |  |  | 8680 | -0.110 | -0.6053 | Yes |
| 251 | ADAM19 |  |  | 8683 | -0.110 | -0.6027 | Yes |
| 252 | RGS3 |  |  | 8684 | -0.110 | -0.6000 | Yes |
| 253 | VIM |  |  | 8691 | -0.110 | -0.5979 | Yes |
| 254 | LRP10 |  |  | 8697 | -0.111 | -0.5957 | Yes |
| 255 | LEPROT |  |  | 8698 | -0.111 | -0.5929 | Yes |
| 256 | IFITM3 |  |  | 8720 | -0.113 | -0.5924 | Yes |
| 257 | GJA1 |  |  | 8728 | -0.114 | -0.5904 | Yes |
| 258 | QSOX1 |  |  | 8731 | -0.114 | -0.5877 | Yes |
| 259 | DDR2 |  |  | 8735 | -0.115 | -0.5852 | Yes |
| 260 | SLC38A2 |  |  | 8750 | -0.117 | -0.5838 | Yes |
| 261 | WWTR1 |  |  | 8752 | -0.117 | -0.5810 | Yes |
| 262 | SSR1 |  |  | 8765 | -0.118 | -0.5794 | Yes |
| 263 | JAM3 |  |  | 8782 | -0.120 | -0.5782 | Yes |
| 264 | THBS1 |  |  | 8789 | -0.120 | -0.5758 | Yes |
| 265 | SQSTM1 |  |  | 8790 | -0.121 | -0.5728 | Yes |
| 266 | PLK2 |  |  | 8803 | -0.123 | -0.5711 | Yes |
| 267 | PLPP1 |  |  | 8821 | -0.125 | -0.5698 | Yes |
| 268 | FLNA |  |  | 8824 | -0.126 | -0.5669 | Yes |
| 269 | TPBG |  |  | 8829 | -0.127 | -0.5642 | Yes |
| 270 | NQO1 |  |  | 8837 | -0.127 | -0.5618 | Yes |
| 271 | ASPH |  |  | 8843 | -0.128 | -0.5592 | Yes |
| 272 | MYDGF |  |  | 8844 | -0.128 | -0.5560 | Yes |
| 273 | PLEKHH2 |  |  | 8850 | -0.129 | -0.5533 | Yes |
| 274 | CD164 |  |  | 8864 | -0.132 | -0.5514 | Yes |
| 275 | ANTXR1 |  |  | 8869 | -0.132 | -0.5486 | Yes |
| 276 | ANXA2 |  |  | 8891 | -0.134 | -0.5476 | Yes |
| 277 | PDIA4 |  |  | 8894 | -0.134 | -0.5444 | Yes |
| 278 | CYR61 |  |  | 8901 | -0.136 | -0.5417 | Yes |
| 279 | FNDC3B |  |  | 8902 | -0.136 | -0.5383 | Yes |
| 280 | DNAJC10 |  |  | 8908 | -0.137 | -0.5354 | Yes |
| 281 | TGFBR2 |  |  | 8916 | -0.137 | -0.5328 | Yes |
| 282 | RCN1 |  |  | 8929 | -0.138 | -0.5307 | Yes |
| 283 | TMEM50A |  |  | 8961 | -0.143 | -0.5305 | Yes |
| 284 | TNFRSF1A |  |  | 8962 | -0.143 | -0.5269 | Yes |
| 285 | TM9SF2 |  |  | 8969 | -0.145 | -0.5240 | Yes |
| 286 | PTGFRN |  |  | 8976 | -0.145 | -0.5210 | Yes |
| 287 | SVIL |  |  | 8981 | -0.146 | -0.5178 | Yes |
| 288 | CDH11 |  |  | 8983 | -0.146 | -0.5143 | Yes |
| 289 | NPTN |  |  | 8985 | -0.146 | -0.5108 | Yes |
| 290 | PTN |  |  | 8989 | -0.147 | -0.5074 | Yes |
| 291 | FZD7 |  |  | 8990 | -0.147 | -0.5037 | Yes |
| 292 | PPIB |  |  | 8991 | -0.147 | -0.5001 | Yes |
| 293 | KDM5B |  |  | 8992 | -0.147 | -0.4964 | Yes |
| 294 | ATXN1 |  |  | 8996 | -0.148 | -0.4930 | Yes |
| 295 | SDC2 |  |  | 9002 | -0.149 | -0.4898 | Yes |
| 296 | PTPN14 |  |  | 9025 | -0.153 | -0.4885 | Yes |
| 297 | F2R |  |  | 9026 | -0.153 | -0.4846 | Yes |
| 298 | MAML2 |  |  | 9028 | -0.154 | -0.4809 | Yes |
| 299 | PON2 |  |  | 9030 | -0.154 | -0.4772 | Yes |
| 300 | FGFR1 |  |  | 9034 | -0.155 | -0.4736 | Yes |
| 301 | WNT5A |  |  | 9037 | -0.156 | -0.4700 | Yes |
| 302 | SERPINH1 |  |  | 9054 | -0.159 | -0.4678 | Yes |
| 303 | LRRC17 |  |  | 9058 | -0.159 | -0.4641 | Yes |
| 304 | TFPI |  |  | 9060 | -0.159 | -0.4602 | Yes |
| 305 | COL4A2 |  |  | 9069 | -0.161 | -0.4571 | Yes |
| 306 | EPHA3 |  |  | 9077 | -0.162 | -0.4538 | Yes |
| 307 | IL6ST |  |  | 9090 | -0.164 | -0.4511 | Yes |
| 308 | MRC2 |  |  | 9106 | -0.167 | -0.4486 | Yes |
| 309 | IGF2R |  |  | 9107 | -0.167 | -0.4444 | Yes |
| 310 | BOC |  |  | 9110 | -0.168 | -0.4404 | Yes |
| 311 | TMBIM4 |  |  | 9112 | -0.169 | -0.4363 | Yes |
| 312 | COL6A2 |  |  | 9116 | -0.170 | -0.4324 | Yes |
| 313 | PPIC |  |  | 9122 | -0.171 | -0.4287 | Yes |
| 314 | SURF4 |  |  | 9123 | -0.171 | -0.4244 | Yes |
| 315 | PTPRG |  |  | 9127 | -0.172 | -0.4205 | Yes |
| 316 | ROBO1 |  |  | 9142 | -0.176 | -0.4176 | Yes |
| 317 | COL5A1 |  |  | 9148 | -0.177 | -0.4138 | Yes |
| 318 | DNAJC3 |  |  | 9150 | -0.177 | -0.4094 | Yes |
| 319 | PRDX4 |  |  | 9151 | -0.178 | -0.4050 | Yes |
| 320 | PRCP |  |  | 9153 | -0.178 | -0.4007 | Yes |
| 321 | EMP1 |  |  | 9156 | -0.179 | -0.3964 | Yes |
| 322 | FSTL1 |  |  | 9158 | -0.179 | -0.3921 | Yes |
| 323 | SRPX |  |  | 9161 | -0.179 | -0.3878 | Yes |
| 324 | PDIA6 |  |  | 9165 | -0.180 | -0.3837 | Yes |
| 325 | ERBIN |  |  | 9166 | -0.180 | -0.3792 | Yes |
| 326 | WLS |  |  | 9168 | -0.181 | -0.3748 | Yes |
| 327 | ATP6V0E1 |  |  | 9173 | -0.182 | -0.3707 | Yes |
| 328 | HTRA1 |  |  | 9178 | -0.184 | -0.3665 | Yes |
| 329 | SYPL1 |  |  | 9182 | -0.185 | -0.3622 | Yes |
| 330 | SERPINE2 |  |  | 9186 | -0.186 | -0.3579 | Yes |
| 331 | PLS3 |  |  | 9187 | -0.186 | -0.3533 | Yes |
| 332 | FAM3C |  |  | 9190 | -0.187 | -0.3488 | Yes |
| 333 | SCRG1 |  |  | 9200 | -0.191 | -0.3451 | Yes |
| 334 | FAT1 |  |  | 9207 | -0.193 | -0.3409 | Yes |
| 335 | DDOST |  |  | 9213 | -0.198 | -0.3365 | Yes |
| 336 | LMAN1 |  |  | 9216 | -0.199 | -0.3317 | Yes |
| 337 | GSN |  |  | 9238 | -0.206 | -0.3289 | Yes |
| 338 | SEMA3C |  |  | 9242 | -0.209 | -0.3240 | Yes |
| 339 | GRN |  |  | 9251 | -0.214 | -0.3196 | Yes |
| 340 | ID3 |  |  | 9252 | -0.214 | -0.3142 | Yes |
| 341 | CTSB |  |  | 9255 | -0.215 | -0.3091 | Yes |
| 342 | CALU |  |  | 9278 | -0.223 | -0.3059 | Yes |
| 343 | COL4A1 |  |  | 9284 | -0.226 | -0.3009 | Yes |
| 344 | ADAM9 |  |  | 9285 | -0.226 | -0.2952 | Yes |
| 345 | LAMC1 |  |  | 9286 | -0.226 | -0.2896 | Yes |
| 346 | RRBP1 |  |  | 9287 | -0.227 | -0.2839 | Yes |
| 347 | PDIA3 |  |  | 9297 | -0.230 | -0.2791 | Yes |
| 348 | LAMB1 |  |  | 9302 | -0.231 | -0.2738 | Yes |
| 349 | TMED9 |  |  | 9305 | -0.233 | -0.2682 | Yes |
| 350 | MMP2 |  |  | 9307 | -0.235 | -0.2625 | Yes |
| 351 | PXDN |  |  | 9312 | -0.240 | -0.2569 | Yes |
| 352 | ATP2B4 |  |  | 9314 | -0.243 | -0.2510 | Yes |
| 353 | COL5A2 |  |  | 9316 | -0.244 | -0.2450 | Yes |
| 354 | LAMP1 |  |  | 9317 | -0.244 | -0.2389 | Yes |
| 355 | GDF15 |  |  | 9322 | -0.247 | -0.2332 | Yes |
| 356 | STAT3 |  |  | 9325 | -0.249 | -0.2272 | Yes |
| 357 | HSP90B1 |  |  | 9334 | -0.257 | -0.2216 | Yes |
| 358 | SYNJ2 |  |  | 9337 | -0.259 | -0.2154 | Yes |
| 359 | ITGAV |  |  | 9346 | -0.269 | -0.2096 | Yes |
| 360 | CD59 |  |  | 9358 | -0.283 | -0.2037 | Yes |
| 361 | SPARCL1 |  |  | 9360 | -0.288 | -0.1966 | Yes |
| 362 | FN1 |  |  | 9364 | -0.294 | -0.1896 | Yes |
| 363 | LAPTM4A |  |  | 9368 | -0.299 | -0.1825 | Yes |
| 364 | B2M |  |  | 9383 | -0.331 | -0.1758 | Yes |
| 365 | SPARC |  |  | 9384 | -0.332 | -0.1675 | Yes |
| 366 | TIMP1 |  |  | 9387 | -0.334 | -0.1593 | Yes |
| 367 | ITGB1 |  |  | 9388 | -0.339 | -0.1509 | Yes |
| 368 | TMEFF2 |  |  | 9390 | -0.347 | -0.1423 | Yes |
| 369 | CALD1 |  |  | 9391 | -0.348 | -0.1336 | Yes |
| 370 | CD63 |  |  | 9394 | -0.353 | -0.1250 | Yes |
| 371 | ITM2B |  |  | 9399 | -0.371 | -0.1162 | Yes |
| 372 | TNC |  |  | 9403 | -0.383 | -0.1070 | Yes |
| 373 | VCL |  |  | 9405 | -0.389 | -0.0974 | Yes |
| 374 | HSPA5 |  |  | 9406 | -0.395 | -0.0875 | Yes |
| 375 | CD44 |  |  | 9407 | -0.397 | -0.0776 | Yes |
| 376 | COL6A1 |  |  | 9410 | -0.410 | -0.0676 | Yes |
| 377 | DKK3 |  |  | 9411 | -0.414 | -0.0573 | Yes |
| 378 | APP |  |  | 9412 | -0.426 | -0.0466 | Yes |
| 379 | CTSC |  |  | 9413 | -0.431 | -0.0359 | Yes |
| 380 | POSTN |  |  | 9437 | -0.750 | -0.0197 | Yes |
| 381 | IGFBP5 |  |  | 9438 | -0.797 | 0.0002 | Yes |
Table: GSEA details [plain text format]

  

Fig 2: VANGRONINGEN\_MES: Random ES distribution      
 Gene set null distribution of ES for **VANGRONINGEN\_MES**
